# Cooperative Assembly of Asymmetric Carbonaceous Bivalve-Like Superstructures from Multiple Building Blocks

**DOI:** 10.1155/2018/5807980

**Published:** 2018-09-02

**Authors:** Lei Xie, Haiyan Wang, Chunhong Chen, Shanjun Mao, Yiqing Chen, Haoran Li, Yong Wang

**Affiliations:** Advanced Materials and Catalysis Group, Institute of Catalysis, Zhejiang University, Hangzhou 310028, China

## Abstract

The assembly of superstructures from building blocks is of fundamental importance for engineering materials with distinct morphologies and properties, and deepening our understanding of self-assembly processes in nature. Up to now, it is still a great challenge in materials science to construct multiple-component superstructure with unprecedented architectural complexity and symmetry from molecular. Here, we demonstrate an improved one-pot hydrothermal carbonization of biomass strategy that is capable of fabricating unprecedented asymmetric carbonaceous bivalve-like superstructures with in suit generated solid particles and ordered porous polymers as two kinds of building blocks. In our system, different building blocks can be controllably generated, and they will assemble into complex superstructures through a proposed “cooperative assembly of particles and ordered porous polymers” mechanism. We believe that this assembly principle will open up new potential fields for the synthesis of superstructures with diverse morphologies, compositions, and properties.

## 1. Introduction

Organized assembly of simple building blocks into complex superstructures is of both scientific and technological importance for designing materials with specific morphologies and distinct properties [1–3]. Such materials are of interest to a variety of fields such as drug delivery [4], energy storage [5], gas adsorption [6], and chemical sensing [7]. Furthermore, superstructuring provides us with an approach to deepen our understanding of self-assembly processes in nature, which occur on molecular to macroscopic scales [8, 9]. To date, a wide variety of methods has been reported to prepare superstructures [10–13]. For example, Mirkin and coworkers introduced a DNA-programmable assembly strategy to assemble triangular bipyramids into clathrate architectures [10]. Klajn and coworkers fabricated helical superstructures from cubic magnetite nanocrystals in the presence of a magnetizing field [13]. However, most of them involve only one kind of building block and require multiple steps (general processes: generation, surface treatment, and assembly of building blocks). The current assembly methods depend not only on the available building blocks (including shape, size, and composition) but also on selective interactions between them and/or external physical factors (e.g., Van der Waals force, electrostatic force, and magnetic interaction) [14, 15]. The major synthetic obstacle to shaped superstructures from multiple building blocks arises from both the complexity of system and the difficulty in controlling assembly, let alone one-pot methods that synthesize and assemble building blocks synchronously [16]. Based on our knowledge, one-pot construction of multiple-component superstructures with unprecedented morphology and symmetry from molecular remains a big challenge in materials science, especially in the hydrothermal carbonization (HTC) carbonaceous materials field.

HTC of biomass in material synthesis was established around a century ago, which is usually applied at mild temperatures (130–250°C) and in aqueous medium inside closed recipients and self-generated pressure [17, 18]. Compared with other routes to fabricate carbonaceous materials, one of the main advantages of one-pot HTC is that it can successfully exploit cheap and environmentally friendly renewable biomass as carbon precursors [19]. However, at the same time, regulating the morphology of product becomes more difficult in comparison with other precursors (such as phenol formaldehyde resin and dopamine) [20–23], due to the complexity of chemical reactions involved in the hydrothermal process of biomass [18]. Although great progress has been made recently due to the unveiling of the HTC-derived carbons structure and their formation mechanism [19, 24–28], to the best of our knowledge, only one spherical superstructure with the introduction of acrylic acid was reported [29]. More complex superstructure assembled from multiple building blocks is still not achieved and remains a challenge.

Here, we demonstrate unprecedented two-component asymmetric carbonaceous bivalve-like superstructures (ACBSs) prepared with an improved one-pot HTC of carbohydrates strategy. Additionally, a “cooperative assembly of particles and ordered porous polymers” formation mechanism is proposed. In our system, xylose was used as carbon precursor, triblock copolymer Pluronic F127 (*EO*_106_-*PO*_70_-*EO*_106_,* Mw = 12600*) and poly (4-styrenesulfonic acid-co-maleic acid) sodium salt (PSSMA) were used as structure-directing agents, and sulfuric acid was used as both a catalyst and a mediator. This strategy enables the controlled generation of carbonaceous solid particles and ordered porous polymers (OPPs) as two kinds of building blocks. Moreover, their cooperative assembly results in complex ACBSs as solid particles tend to aggregate to spherical clusters and OPPs are inclined to form hexagonal morphology coated on particles. Owing to this unique bivalve-like morphology with large-sized opening and micro-ordered pore structure, bivalve-like carbon superstructures after subsequent carbonization have shown good performance of supercapacitors. Moreover, we speculate that this assembly principle would dramatically expand the variety of superstructures and create unprecedented architectures.

## 2. Results

### 2.1. Structural Characteristics

**Figure 1:**
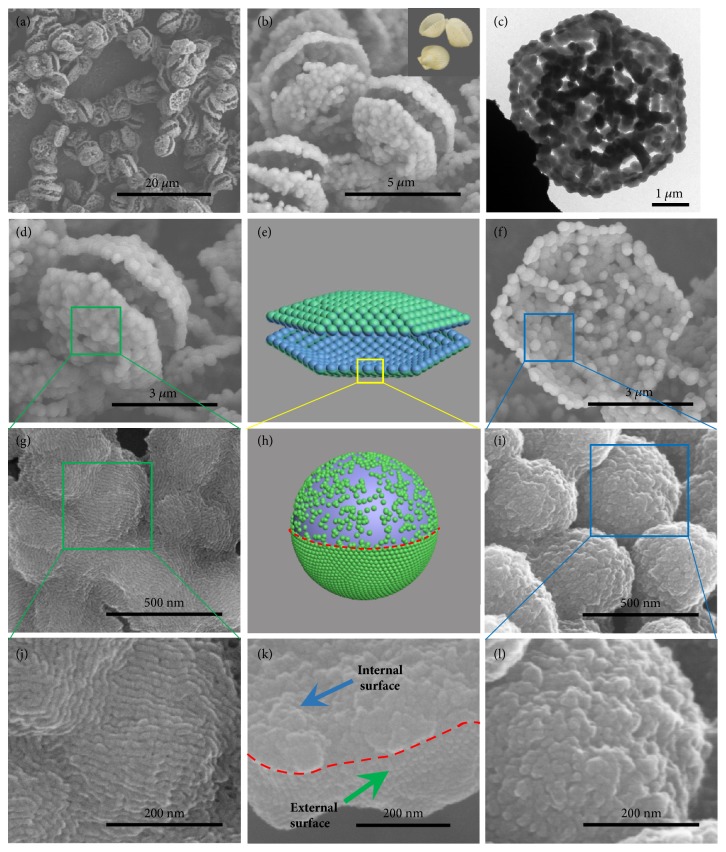
**Structural characteristics of ACBSs.** (**a, b**) SEM images of ACBSs at low magnification, digital photograph of the bivalves (inside (b)). (**c**) TEM image of ACBSs at low magnification. SEM images of ACBSs: (**d, g, j**) external surface, (**f, i, l**) internal surface. (**e, h**) ACBSs model with a green external surface, a blue internal surface, and a red boundary. (**k**) Sectional surface: red line shows the boundary of external and internal surfaces.

Asymmetric carbonaceous bivalve-like superstructures (ACBSs) were synthesized through hydrothermal reaction of xylose at 140°C for 4.0 h in the presence of sulfuric acid, and triblock copolymer Pluronic F127 and PSSMA were used as structure-directing agents. Scanning electron microscopy (SEM) images show that ACBSs are homogenous in large area (Figure 1(a), [Sec supplementary-material-1]) and composed of two linked and hexagonal platelets with slight curvature (Figure 1(b), [Sec supplementary-material-1]), which is similar to natural bivalves (inside Figure 1(b)). Moreover, the approximately 5 *μ*m hexagonal shell is made up of about 300 nm particles, as confirmed by transmission electron microscopy (TEM) images (Figure 1(c), [Sec supplementary-material-1]).

Interestingly, close observation at a higher magnification reveals that ordered porous polymers (OPPs) coat on the external surface of ACBSs (Figures 1(d), 1(g), and 1(j)), which is in line with magnified TEM image ([Sec supplementary-material-1]). In contrast, polymers coated on the internal surface arrange irregularly (Figures 1(f), 1(i), and 1(l)). The boundary between these two surfaces is shown in Figures 1(k) and 1(e) (red line), as illustrated in the ACBSs model with a green external surface and a blue internal surface (Figures 1(h) and 1(k)). Another character is that the granular outline of the internal surface (Figures 1(d), 1(g), and 1(j)) is much clearer than that of the external surface (Figures 1(f), 1(i), and 1(l)), indicating that there are more polymers coated on the external surface, which is confirmed by sliced high-resolution TEM image ([Sec supplementary-material-1]).

### 2.2. Formation Process

**Figure 2:**
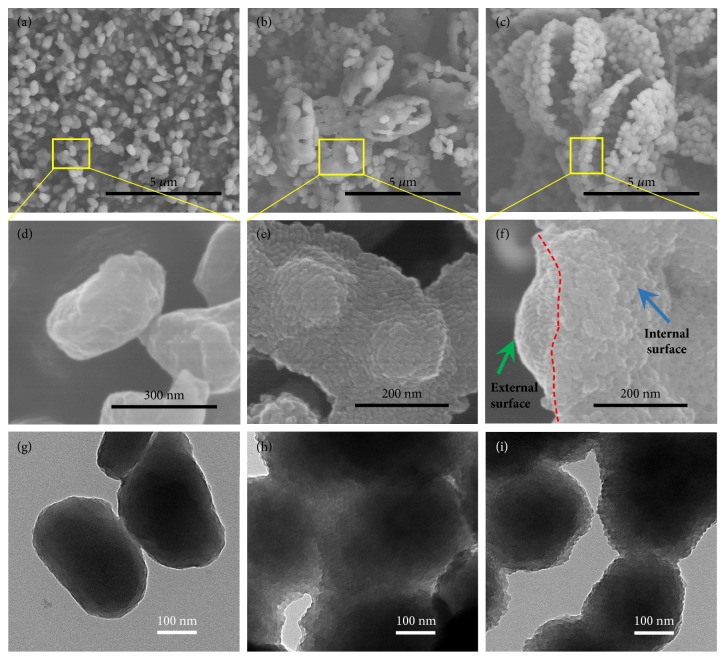
**Formation process of ACBSs.** SEM and TEM micrographs of samples at different reaction times. (**a, d, g**) Solid particles at 1.0 h.** (b, e, h)** Small bilaminar plates assembled form particles at 2.0 h, with OPPs coating on these particles. (**c, f, i**) ACBSs at 4.0 h.

To gain insights into the shape evolution and the possible formation mechanism of ACBSs, we monitored their time-dependent formation behavior by SEM and TEM. As shown in Figure 2, solid particles formed at an early stage (1.0 h, Figures 2(a), 2(d), and 2(g)). As the hydrothermal reaction time extended, significant distinctions appeared: solid particles aggregated into small bilaminar plates, accompanied by OPPs coating on those particles (2.0 h, Figures 2(b), 2(e), and 2(h)). They have continuously grown to larger dehiscent bilaminar hexagons, that is, ACBSs, with a prolonged reaction time (4.0 h, Figures 2(c), 2(f), and 2(i)). Yields at various times indicate that abundant solid particles formed quickly in 2.0 h, and OPPs appeared slowly later ([Sec supplementary-material-1]). In short, solid particles and OPPs coexisted in our system, and they assembled into small bilaminar hexagons and further into ACBSs as the reaction progressed.

### 2.3. Controlled Generation of Solid Particles and OPPs

**Figure 3:**
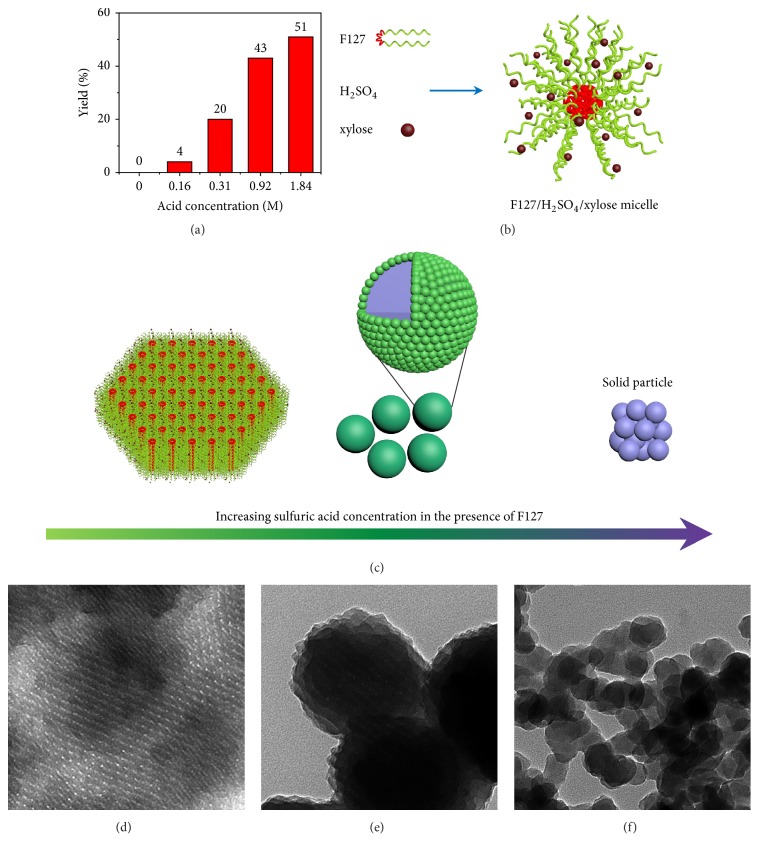
**The functions of sulfuric acid and F127.** (**a**) Yields with various acid concentrations. (**b**) Model of F127/H_2_SO_4_/xylose composite micelle. (**c**) Schematic illustration of products at different acid concentrations in the presence of F127, from OPPs to solid particles. TEM images of products with different acid concentrations in the presence of F127: (**d**) 0.92 M, (**e**) 1.53 M, (**f**) 1.84 M.

To elucidate the formation mechanism, a series of supplementary experiments were conducted. Sulfuric acid applied in our system was investigated firstly. On the one hand, high hydrothermal yields were obtained when sulfuric acid was introduced into xylose solution (Figure 3(a)), while no product was obtained without acid at such a low temperature (140°C) and during such a short reaction time (4.0 h). Thus, we conclude that sulfuric acid acts as an effective hydrothermal catalyst that can accelerate the hydrolysis and polymerization rate of xylose [30]. Moreover, only irregular and solid particles formed with various acid concentrations (Figures [Sec supplementary-material-1]-F), even at different reaction times (Figures [Sec supplementary-material-1]-I). On the other hand, Pluronic F127, containing both hydrophilic groups (PEO chains) and hydrophobic groups (PPO chains), will aggregate to micelles as the concentration employed in our system was much higher than the critical micelle concentration [31]. Dynamic lighting scatting results ([Sec supplementary-material-1]) indicate that acid caused a notable increase of micellar size from 6 to 18 nm in the presence of F127 and xylose. This size increase occurs because sulfuric acid protonates F127 and xylose, and both hydrogen bond and coulombic interaction subsequently drive the self-assembly of F127 and xylose into the enhanced stable structure—F127/H_2_SO_4_/xylose composite micelles (Figure 3(b), [Sec supplementary-material-1]), which is similar to the S^0^H^+^X^−^I^+^ self-assembly mechanism [32]. According to the previous report, the assembly of micelles and polymerization of carbohydrates will give rise to OPPs [33].

Furthermore, as shown in Figure 3 and [Sec supplementary-material-1], the formation of solid particles and OPPs can be regulated through the variation of the sulfuric acid concentration in the presence of F127 (only without PSSMA compared to the formation of ACBSs). For lower acid concentration, abundant micelles can exist stably to anisotropically form OPPs through hexagonal p6mm self-assembly (Figure 3(d), Figures [Sec supplementary-material-1] and D) [34]. At acid concentration for generating ACBSs, two building blocks coexisted in the same system (Figure 3(e), Figures [Sec supplementary-material-1] and E). In order to give a further insight to the effect of F127, samples at different times were prepared ([Sec supplementary-material-1]). Similar to the formation process of ACBSs, massive solid particles (Figures [Sec supplementary-material-1], D, and G) that were much smaller than those obtained with only acid added ([Sec supplementary-material-1]) appeared in the early stage, because F127 can act as a surfactant to stabilize them. Then OPPs from the assembly of micelles and the polymerization of carbohydrates coated on these solid particles (Figures [Sec supplementary-material-1], E, and H), whereas only irregular structure finally formed (Figure 3(e), Figures [Sec supplementary-material-1] and E, Figures [Sec supplementary-material-1], F, and I). With a further increase of acid concentration, faster hydrolysis and polymerization rate of xylose will lead to the formation of only solid particles (Figure 3(f), Figures [Sec supplementary-material-1] and F). Moreover, the nanostructures of these carbon materials after carbonization were further examined with nitrogen sorption characterization ([Sec supplementary-material-1]). The ordered mesoporous structure of samples with a low acid concentration was confirmed by the type-IV isotherm and the pore-size distribution using the Barrett−Joyner−Halenda model (Figures [Sec supplementary-material-1] and D). As acid content increased to 1.53M, the less mesopores could also be indicated (Figures [Sec supplementary-material-1] and E). With a further increase of acid, the N_2_ sorption isotherms of product exhibit type I curve with a hysteresis loop at high relative pressure, which is typically associated with mesopores caused by interparticle voids and micropores (Figures [Sec supplementary-material-1] and F). Briefly, solid particles and/or OPPs can be generated by F127 and sulfuric acid, and they can coexist in our system.

### 2.4. Assembly Types of Solid Particles and OPPs

**Figure 4:**
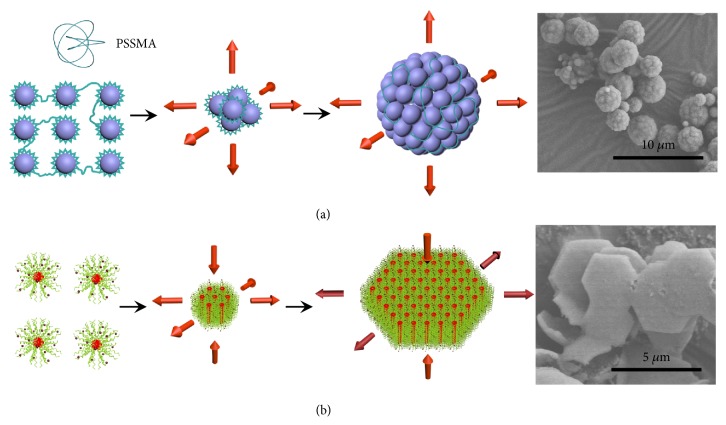
**Images and schematic illustration of the formation of spherical clusters and hexagonal OPPs. **(**a**) Schematic illustration and SEM image of the formation of spherical clusters with PSSMA and acid. (**b**) Schematic illustration and SEM image of the formation of hexagonal OPPs from F127/H_2_SO_4_/xylose composite micelles with F127 and 0.92 M acid.

As there are two building blocks in this process, the assembly styles of them will play crucial role in the formation of ACBSs. PSSMA, which is widely used as a stabilizer for the synthesis of a variety of water-soluble nanomaterials [35, 36], was investigated firstly. Samples with the addition of PSSMA in the presence of acid (only without F127 compared to the formation of ACBSs) were prepared at different reaction times ([Sec supplementary-material-1]). Particles at 1.0 h ([Sec supplementary-material-1]) were much smaller than that with only acid added ([Sec supplementary-material-1]), indicating that PSSMA may attach to the surfaces of particles to lower their surface energy [37, 38]. With the extension of reaction time, these small particles assembled isotropically into dispersive spherical clusters (Figures [Sec supplementary-material-1], C, Figure 4(a)). This phenomenon mainly arises from the instability of small particles, and they tend to aggregate isotropically to further lower their surface energy, which is similar to other demonstrations with the addition of PSSMA [36, 38]. Spherical clusters with a more negative charge will offer stronger electrostatic repulsion between them ([Sec supplementary-material-1]), which leads to a dispersive spherical structure [36]. Therefore, we conclude that, in the presence of sulfuric acid, PSSMA can regulate small particles to assemble isotropically into dispersive spherical clusters (Figure 4(a)). In addition, as mentioned above, OPPs anisotropically formed through hexagonal p6mm self-assembly of micelles exhibit hexagonal morphology ([Sec supplementary-material-1]), which favors 100 orientation growth while 001 face remains stable (Figure 4(b)). We believe this is the main reason for the hexagonal structure of ACBSs because the arrangement types of hexagonal OPPs ([Sec supplementary-material-1]) and ACBSs (Figure 1(j)) are the same.

### 2.5. CAPOPP Mechanism

**Figure 5:**
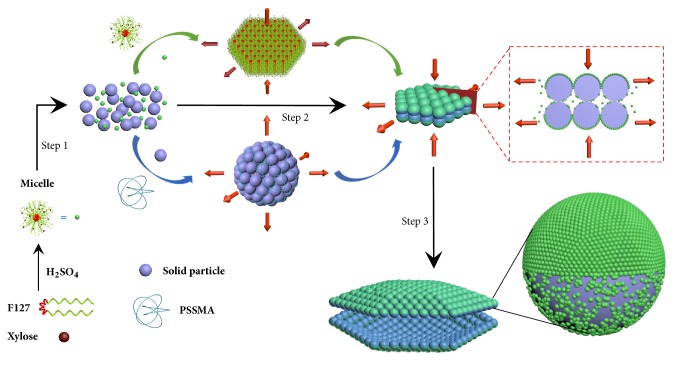
**Schematic illustration of ACBSs formation process.** Step 1, a large number of solid particles and F127/H_2_SO_4_/xylose composite micelles form within a short time. Step 2, cooperative assembly of particles and OPPs from micelles gives rise to small bilaminar plates. Step 3, sustained growth results in ACBSs.

Based on the aforementioned analyses, we believe that the cooperative assembly of these two building blocks leads to the formation of ACBSs, and the postulated “cooperative assembly of particles and ordered porous polymers” (CAPOPP) mechanism is illustrated in Figure 5. In the initial stage (Step 1), fast hydrolysis and polymerization rate of xylose at a high sulfuric acid concentration leads to the formation of a large number of solid particles in a short time; these particles can be stabilized momentarily by F127 and PSSMA. In the second step (Step 2), solid particles tend to isotropically assemble into spherical clusters assisted with PSSMA. At the same time, F127/H_2_SO_4_/xylose composite micelles tend to arrange on the external surfaces of aggregated particles and form hexagonal OPPs via hexagonal p6mm self-assembly, which limits the growth of 001 faces. As a result, cooperative assembly of them gives rise to small hexagonal bilaminar plates. In the last stage (Step 3), sustained growth results in large dehiscent bilaminar hexagons (that is ACBSs). In addition, their external surfaces are coated with abundant OPPs, whereas the internal surfaces are coated with less and disordered polymers because of insufficient contact of micelles and particles, and the asymmetric surfaces may be the reason that drives bilaminar plates to split.

To demonstrate the versatility of this synthesis method, we replace xylose and sulfuric acid with arabinose and hydrochloric, respectively. As shown in Figures [Sec supplementary-material-1] and B, all products exhibit bivalve-like morphologies, which indicates that this strategy is a general route to fabricate ACBSs. Moreover, success of the scale-up experiment indicates that this method can be applied to mass production (Figures [Sec supplementary-material-1] and D).

### 2.6. Electrochemical Performances

**Figure 6:**
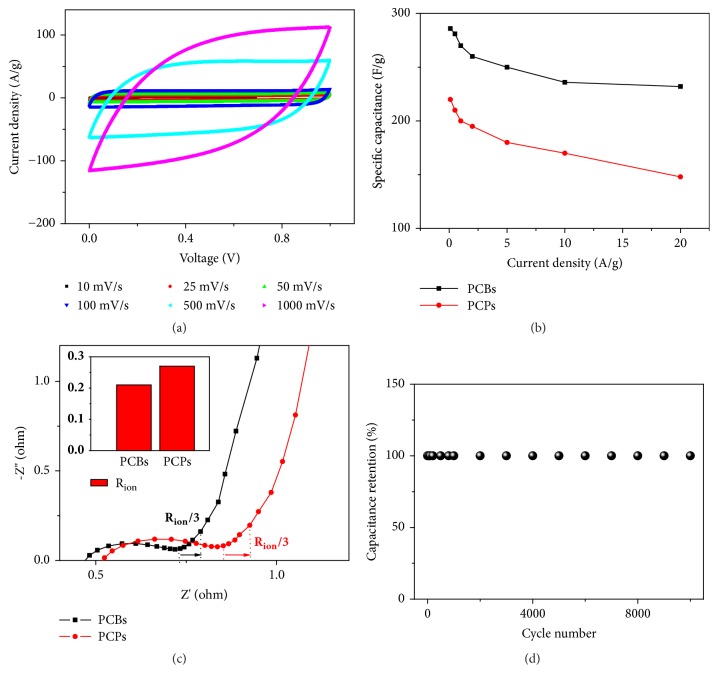
**Electrochemical performances of PCBs and PCPs.** (**a**) CV curves of PCBs at various sweep rates. (**b**) Comparison of specific capacities of PCBs and PCPs at various current densities. (**c**) Comparison of Nyquist plots for PCBs and PCPs. The projection of the 45° slope in the high-frequency region is defined as R_ion_/3, which is utilized to determine the ionic resistance for the electrolyte-filled pores in a nonfaradaic process. (**d**) Cyclic stability of PCBs at 20 A g^−1^ over 10 000 cycles.

Porous carbon bivalves (PCBs) were further obtained by subsequent carbonization of ACBSs at 900°C with the aid of foaming agents ([Sec supplementary-material-1]) [39]. To better reveal the superiority of PCBs, porous carbon particles (PCPs) from irregular structure materials (only without PSSMA compared to the fabrication of ACBSs, Figure 3(e), Figures [Sec supplementary-material-1] and [Sec supplementary-material-1]) were prepared for contrast ([Sec supplementary-material-1]). The specific surface areas for PCBs and PCPs were 1991 and 1680 m^2^ g^−1^, respectively, and the pore-size distribution demonstrates the existence of micropores, mesopores, and macropores (Figures [Sec supplementary-material-1] and D). In addition, the volume of mesopores was calculated to be 0.41 cm^3^ g^−1^ for PCBs, which is larger than that for PCPs. In addition, pore volume distributions measured by mercury porosimetry have shown that there were larger pores of PCBs than PCPs (Figures [Sec supplementary-material-1] and F). Given the unique bivalve-like morphology with large-sized opening for ion-buffering reservoirs and well-organized accumulation with micro-ordered pore structure facilitating rapid ion transport and mitigating diffusion limitations, PCBs show promise for supercapacitors. The supercapacitors performances were then evaluated with a symmetrical two-electrode test system in 6 M KOH electrolyte. The cyclic voltammetry (CV) curves in Figure 6(a) with nearly symmetrical rectangular shapes from 10 mV s^−1^ to 1000 mV s^−1^ manifest the ideal electric double-layer capacitance behavior in PCBs. The maximum specific capacitance of PCBs was calculated to be 286 F g^−1^ at 0.1 A g^−1^, which is not only higher than 220 F g^−1^ for PCPs (Figure 6(b) and [Sec supplementary-material-1]), but also among the best values reported for porous carbon materials obtained from HTC of biomass ([Sec supplementary-material-1]). Additionally, high capacitance retention of 81% was achieved by PCBs with a 200-fold increase in current density, which is better than that of PCPs (67%). The reason for the excellent rate capability can be further probed by analyzing the projection of the 45° slope to the area in the Nyquist plots (Figure 6(c)), which reflects the ionic resistance (R_ion_) for the electrolyte-filled pores inside the electrode structure in a nonfaradaic process [40]. As a result, PCBs show R_ion_ of 0.21 ohm cm^−2^, lower than 0.27 ohm cm^−2^ for PCPs, demonstrating the faster ion transport in the entire PCBs electrode. These results combined with an energy density of 9.93 W h kg^−1^ and a stable cycling performance after 10000 cycles (Figure 6(d)) further promise the application of PCBs for advanced energy storage devices. As the only difference is the bivalve-like structure of PCBs and the disordered structure of PCPs, we believe that the more mesopores and macropores pores and larger specific surface area resulted from this bivalve-like structure enhance the performance of supercapacitors.

## 3. Discussion

In this study, ACBSs assembled form multiple building blocks were fabricated through a “cooperative assembly of particles and ordered porous polymers” mechanism, with a one-pot hydrothermal treatment of biomass method in the presence of two structure-directing agents and sulfuric acid. This simple strategy enables the controlled generation of carbonaceous solid particles and ordered porous polymers as two types of building blocks, and they will further assemble into ACBSs. This asymmetric bivalve-like structure with large-sized opening and micro-ordered pores of carbon porous bivalves enhanced the performance of supercapacitors. We believe that by controlling the kind and self-assembly form of building blocks in this system, a variety of multicomponent superstructures with diverse morphologies and properties can be obtained based on the same principle.

## 4. Materials and Methods

### 4.1. Materials

F127 is purchased from Sigma-Aldrich. Xylose, arabinose, and PSSMA are supplied by Aladdin. Sulfuric acid, hydrochloric acid, (NH_4_)_2_C_2_O_4_·H_2_O (AR), and KHCO_3_ (AR) are purchased from Sinopharm Chemical Reagent Co., Ltd. All chemicals are used as received without any further purification.

### 4.2. Synthesis of ACBSs

In a typical procedure, 6.0 g xylose and 3.0 g F127 are added to 1.53 M acid solution, and the mixture forms transparent solution after stirring for 12 h at room temperature. Then 150 mg PSSMA is added; after stirring for another 12 h, the resultant solution is transferred into 100 mL autoclave and hydrothermally treated at 140°C for 4 h. After the autoclave cools to room temperature, the solid products are collected by filtration, washed three times with water and ethanol, and dried at 70°C overnight.

### 4.3. Synthesis of PCBs and PCPs

Typically, a mixture of ACBSs, (NH_4_)_2_C_2_O_4_·H_2_O, and KHCO_3_ (mass ratio of 1:4:4) is mixed thoroughly by grinding for 30 min. Then the mixture is calcined to 600°C at a heating rate of 10°C min^−1^ and is held at that temperature for 1 h under N_2_ atmosphere. The sample is then further heated to 900°C at a rate of 5°C min^−1^ and kept for 1 h. After the sample is cooled, the black powder was dissolved in an acid aqueous solution and stirred for 12 h. The PCBs are obtained after the powder solution is washed with deionized water several times and dried in an oven overnight. PCPs were synthesized and tested under similar conditions, and we replaced ACBSs with the carbonaceous materials without PSSMA.

### 4.4. Characterization

SEM images are obtained using a Hitachi SU-8010. TEM is carried out with a Hitachi HT-7700 microscope. Dynamic lighting scatting and zeta potential measurements are recorded on a Malvern Zetasizer Nano-ZS using laser radiation with a wavelength of 633 nm and a power of 4 mW. The scattered light is measured at a backscattering angle of 173°. The N_2_ adsorption-desorption isothermal analysis was performed using a Micromeritics ASAP 2020 HD88, and the surface area was calculated using the BET equation. Mercury (Hg) porosimetry is performed with AutoPore IV 9510.

### 4.5. Electrochemical Measurements for Supercapacitors

The electrochemical performances of all the carbon samples were measured in 6 M KOH electrolyte using a symmetric two-electrode testing system. The electrodes were prepared by mixing the carbon samples and polytetrafluoroethylene in the ratio of 9:1. The suspension was pressed onto a nickel foam current collector with the active surface area of 1 cm^2^. The electrodes were then dried and weighed. The mass density of active materials per electrode was approximately 2.5 mg cm^−2^. Two electrodes with identical or close mass were selected for the two-electrode measurements with 6 M KOH as electrolyte. The capacitive performances were evaluated by cyclic voltammetry (CV), galvanostatic charge/discharge (GCD), and electrochemical impedance spectroscopy tests. All the electrochemical measurements were carried out using a Gamry Reference 600 electrochemical workstation at room temperature.

## Data Availability

All data needed to evaluate the conclusions in the paper are present in the paper and/or the Supplementary Materials. Additional data related to this paper may be requested from the authors.

## Supplementary Materials

Supplementary Materials Figure S1: SEM and TEM images of ACBSs. Figure S2: yields of products at different reaction times. Figure S3: SEM and TEM images of materials with different acid concentrations and different reaction times. Figure S4: dynamic lighting scatting and model of F127/H_2_SO_4_/xylose composite micelle. Figure S5: SEM images of materials obtained with F127 at different sulfuric acid concentrations. Figure S6: SEM and TEM images of materials obtained with 1.53 M sulfuric acid and F127 at different reaction times. Figure S7: nitrogen adsorption isotherms and pore-size distribution using the Barrett−Joyner−Halenda (BJH) model of materials obtained with F127 at different sulfuric acid concentrations. Figure S8: SEM images of the formation of spherical clusters with PSSMA and acid at different reaction times. Figure S9: zeta potential of materials at different reaction times. Figure S10: SEM images of materials. Figure S11: characteristics of PCBs and PCPs. Figure S12: GCD curves at various current densities from 0.1 A g^−1^ to 20 A g^−1^. Table S1: summary of the performances of representative porous carbon electrodes from HTC of biomass tested in aqueous electrolyte with a symmetric two-electrode test system.
