# Supplementary material for: Cooperative Assembly of Asymmetric Carbonaceous Bivalve-Like Superstructures from Multiple Building Blocks
Source: Research (Wash D C). 2018 Sep 2;2018:5807980. doi: 10.1155/2018/5807980 (PMC6750077; doi:10.1155/2018/5807980)
Supplement: Supplementary Materials — Figure S1: SEM and TEM images of ACBSs. Figure S2: yields of products at different reaction times. Figure S3: SEM and TEM images of materials with different acid concentrations and different reaction times. Figure S4: dynamic lighting scatting and model of F127/H2SO4/xylose composite micelle. Figure S5: SEM images of materials obtained with F127 at different sulfuric acid concentrations. Figure S6: SEM and TEM images of materials obtained with 1.53 M sulfuric acid and F127 at different reaction times. Figure S7: nitrogen adsorption isotherms and pore-size distribution using the Barrett−Joyner−Halenda (BJH) model of materials obtained with F127 at different sulfuric acid concentrations. Figure S8: SEM images of the formation of spherical clusters with PSSMA and acid at different reaction times. Figure S9: zeta potential of materials at different reaction times. Figure S10: SEM images of materials. Figure S11: characteristics of PCBs and PCPs. Figure S12: GCD curves at various current densities from 0.1 A g−1 to 20 A g−1. Table S1: summary of the performances of representative porous carbon electrodes from HTC of biomass tested in aqueous electrolyte with a symmetric two-electrode test system. [file 5807980.f1.docx]

**Supplementary Information**

**Cooperative assembly of asymmetric carbonaceous bivalve-like superstructures from multiple building blocks**

Lei Xie, Haiyan Wang, Chunhong Chen, Shanjun Mao, Yiqing Chen, Haoran Li, Yong Wang *

Advanced Materials and Catalysis Group, Institute of Catalysis, Zhejiang University, Hangzhou 310028, P. R. China.

*Corresponding author. E-mail: [chemwy@zju.edu.cn](mailto:chemwy@zju.edu.cn)

**Fig. S1.** SEM and TEM images of ACBSs.

**Fig. S2.** Yields of products at different reaction times.

**Fig. S3.** SEM and TEM images of materials with different acid concentrations, and different reaction times.

**Fig. S4.** Dynamic lighting scatting and Model of F127/H_2_SO_4_/xylose composite micelle.

**Fig. S5**. SEM images of materials obtained with F127 at different sulfuric acid concentrations.

**Fig. S6**. SEM and TEM images of materials obtained with1.53 M sulfuric acid and F127 at different reaction times.

**Fig. S7.** Nitrogen adsorption isotherms and pore-size distribution using the Barrett−Joyner−Halenda (BJH) model of materials obtained with F127 at different sulfuric acid concentrations. (A, D) 0.92 M. (B, E) 1.53 M. (C, F) 1.84 M.

**Fig. S8.** SEM images of the formation of spherical clusters with PSSMA and acid at different reaction times.

**Fig. S9**. Zeta potential of materials at different reaction times.

**Fig. S10.** SEM images of materials.

**Fig. S11.** Characteristics OF PCBs and PCPs.

**Fig. S12.** GCD curves at various current densities from 0.1 A g^-1^ to 20 A g^-1^.

**Table S1.** Summary of the performances of representative porous carbon electrodes from HTC of biomass tested in aqueous electrolyte with a symmetric two-electrode test system.


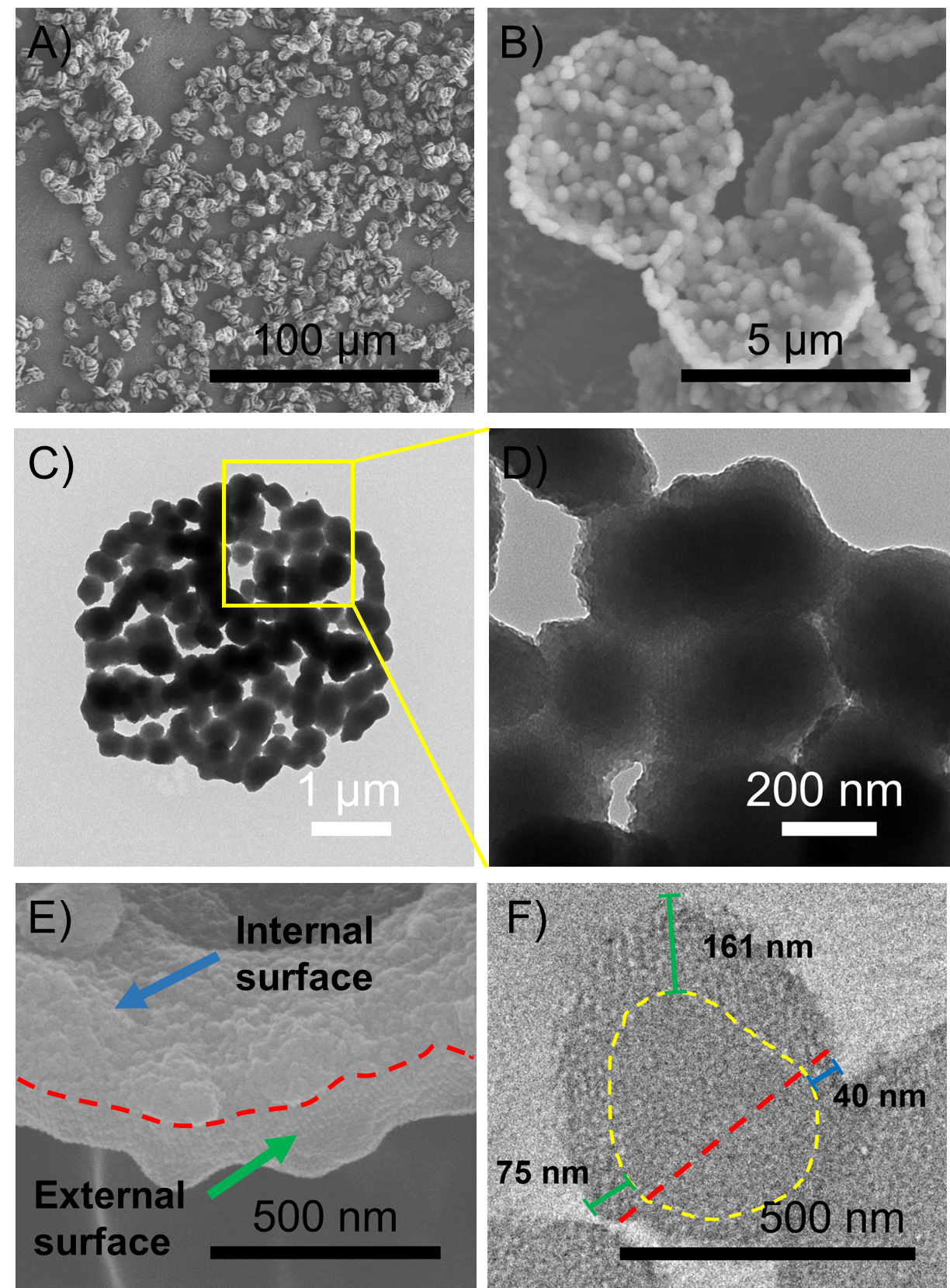


**Fig. S1. SEM and TEM images of ACBSs.** (**A**) SEM image at low magnification. (**B**) SEM image of opened ACBSs. (**C, D**) TEM images. (**E**) Sectional surface, red line shows the boundary of external and internal surfaces. (**F**) Sliced high-resolution TEM image of ACBSs, yellow line shows the outline of solid particle, green lines indicate the thickness of OPPs coated on external surface, blue one demonstrates that on internal surface, and red line means the boundary.

**Fig. S2. Yields of products at different reaction times.**


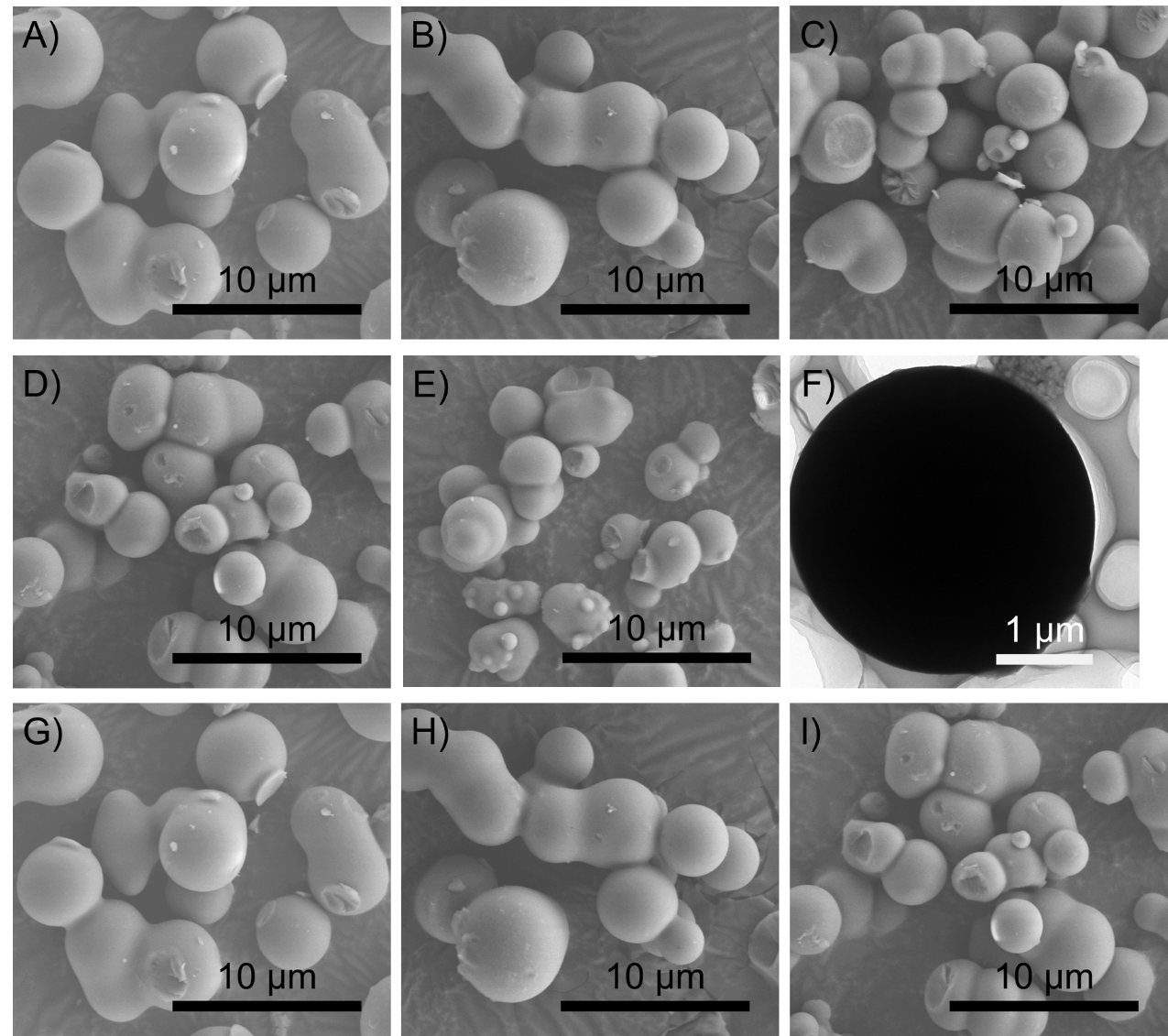


**Fig. S3. SEM and TEM images of materials with different acid concentrations, and different reaction times.** (**A**) 0.15 M. (**B**) 0.31 M. (**C**) 0.62 M. (**D**) 1.53 M. (**E**) 1.84 M. (**F**) TEM image with 1.53 M acid. SEM images of materials obtained with 1.53 M sulfuric acid at different reaction times. (**G**) 1.0 h. (**H**) 2.0 h. (**I**) 4.0 h.


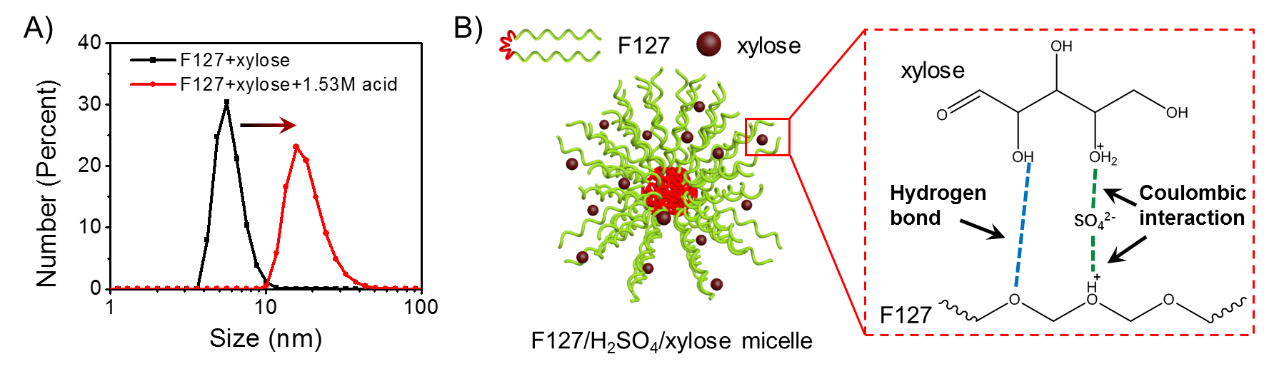


**Fig. S4. Dynamic lighting scatting and Model of F127/H_2_SO_4_/xylose composite micelle.** (**A**) Dynamic lighting scatting of micelles with and without acid. (**B**) Model of F127/H_2_SO_4_/xylose composite micelle with both hydrogen bond and Coulombic interaction.


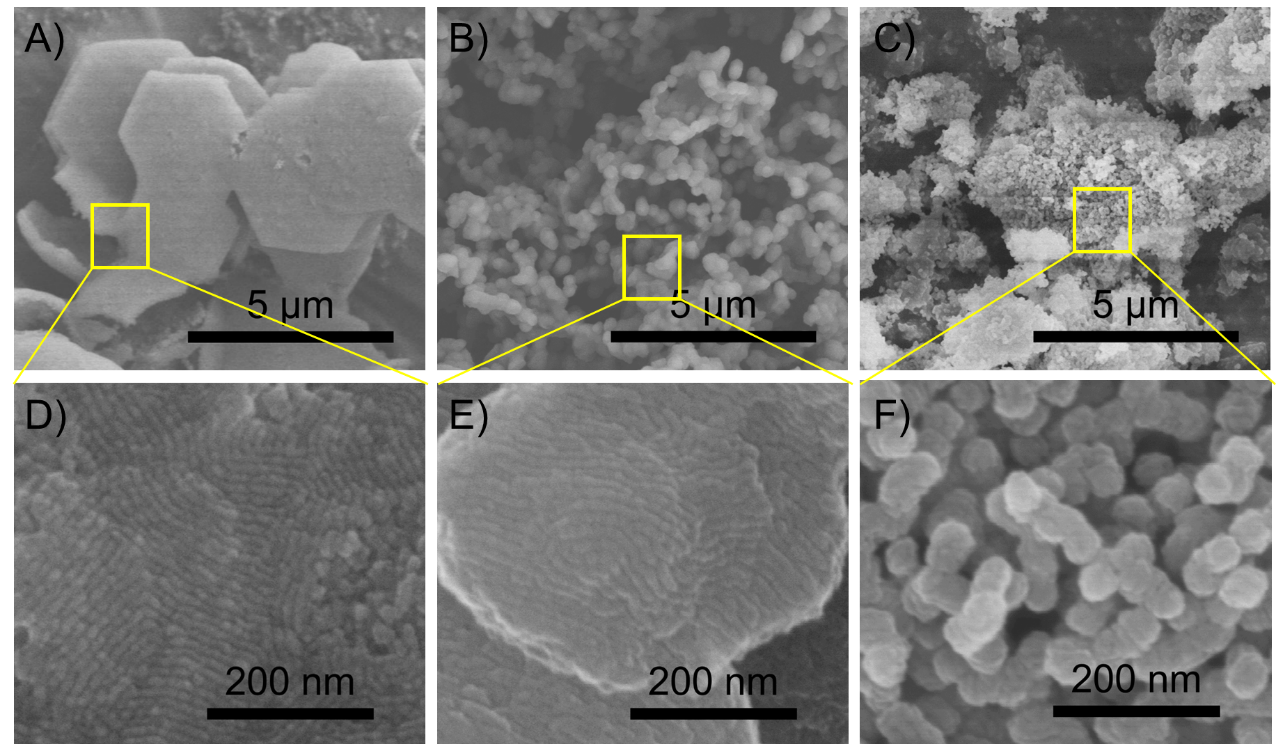


**Fig. S5. SEM images of materials obtained with F127 at different sulfuric acid concentrations.** (**A, D**) 0.92 M. (**B, E**) 1.53 M. (**C, F**) 1.84 M.


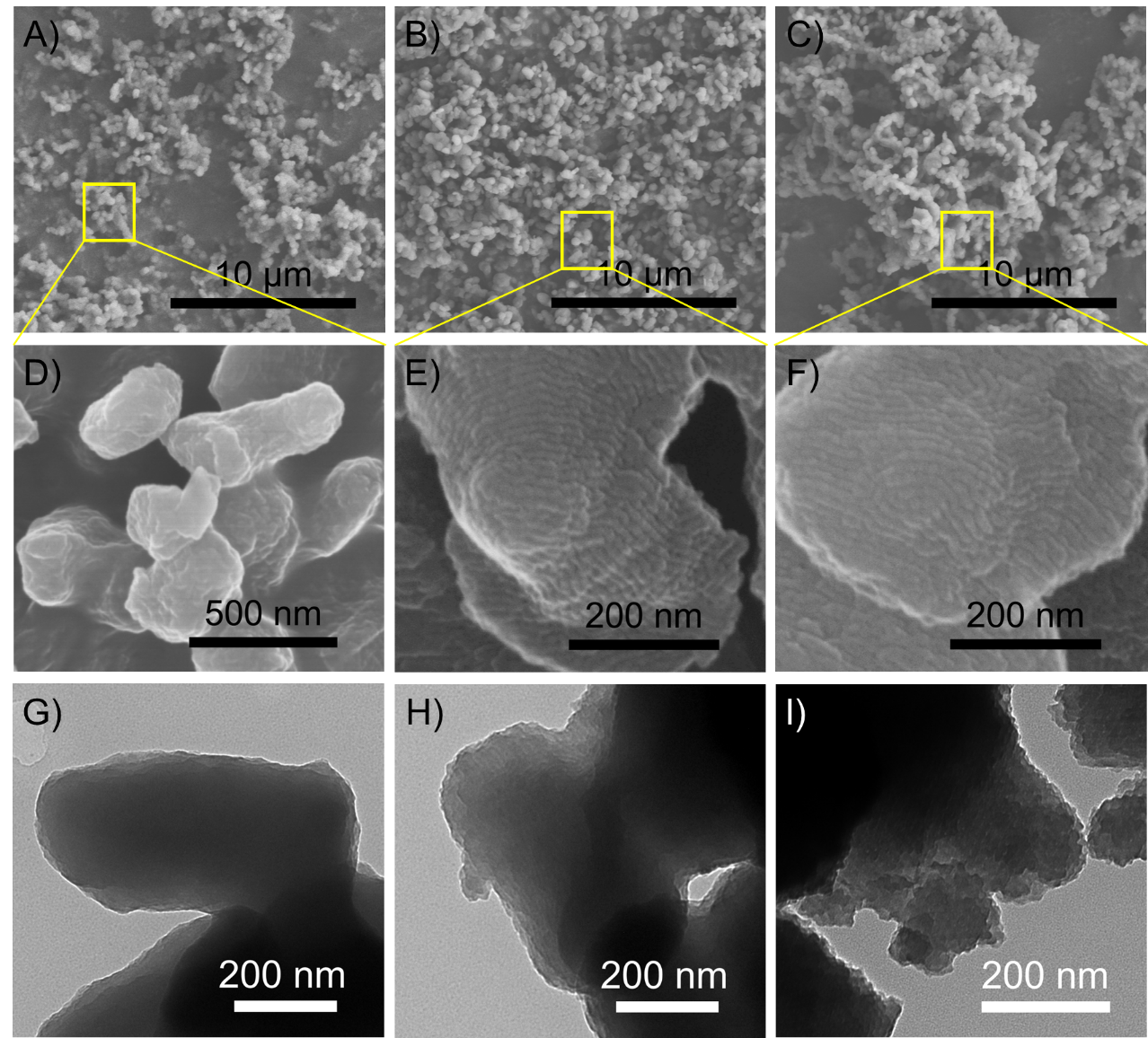


**Fig. S6. SEM and TEM images of materials obtained with1.53 M sulfuric acid and F127 at different reaction times.** (**A, D, G**) 1.0 h. (**B, E, H**) 2.0 h. (**C, F, I**) 4.0 h.


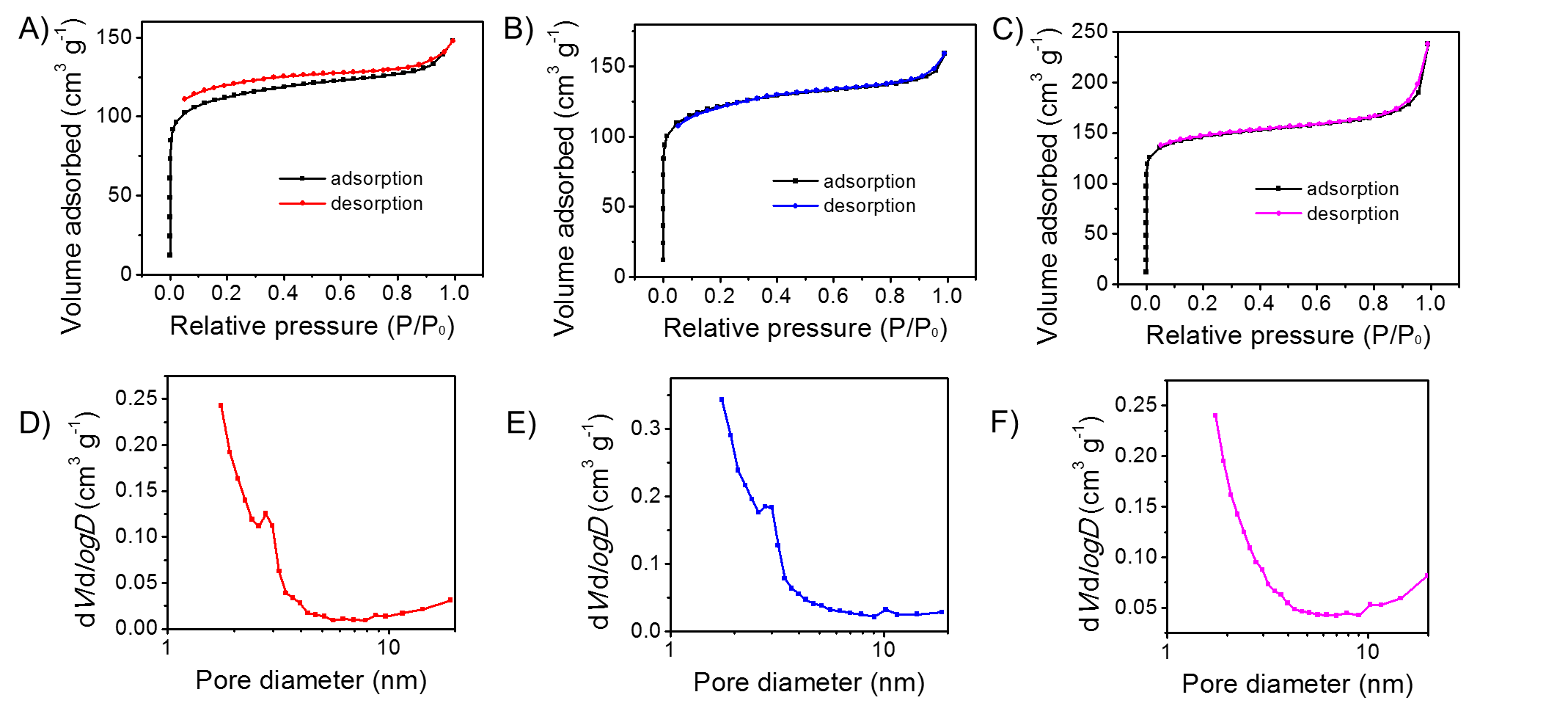


**Fig. S7. Nitrogen adsorption isotherms and pore-size distribution using the Barrett−Joyner−Halenda (BJH) model of materials obtained with F127 at different sulfuric acid concentrations.** (**A, D**) 0.92 M. (**B, E**) 1.53 M. (**C, F**) 1.84 M.


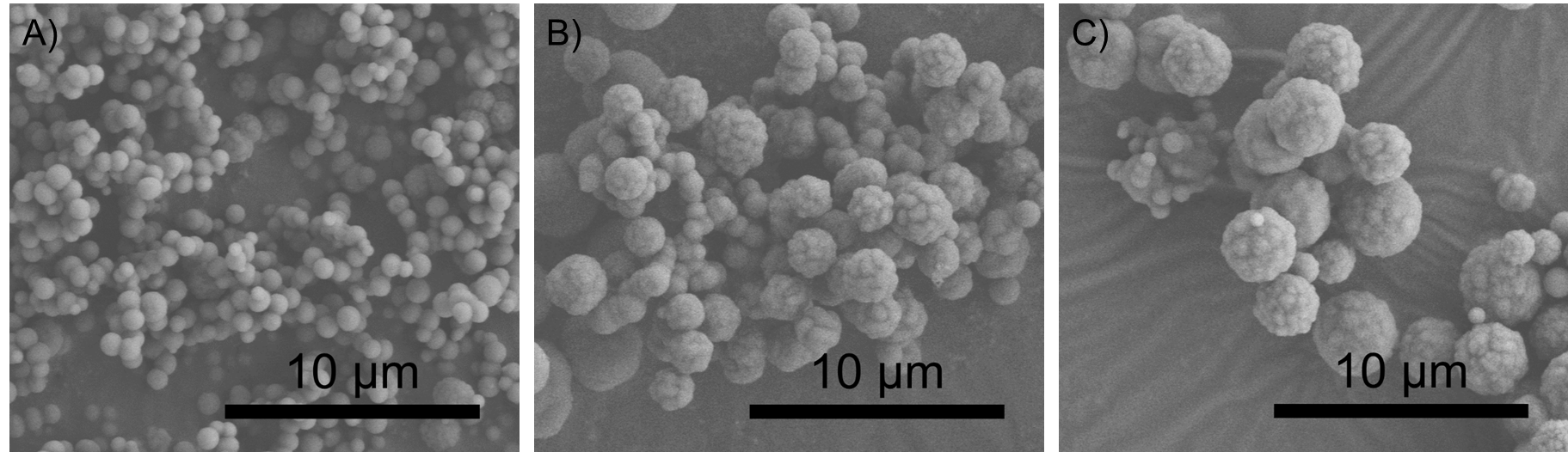


**Fig. S8. SEM images of the formation of spherical clusters with PSSMA and acid at different reaction times: (A)** 1.0 h, **(B)** 2.0 h, **(C)** 4.0 h.

**Fig. S9. Zeta potential of materials at different reaction times.**


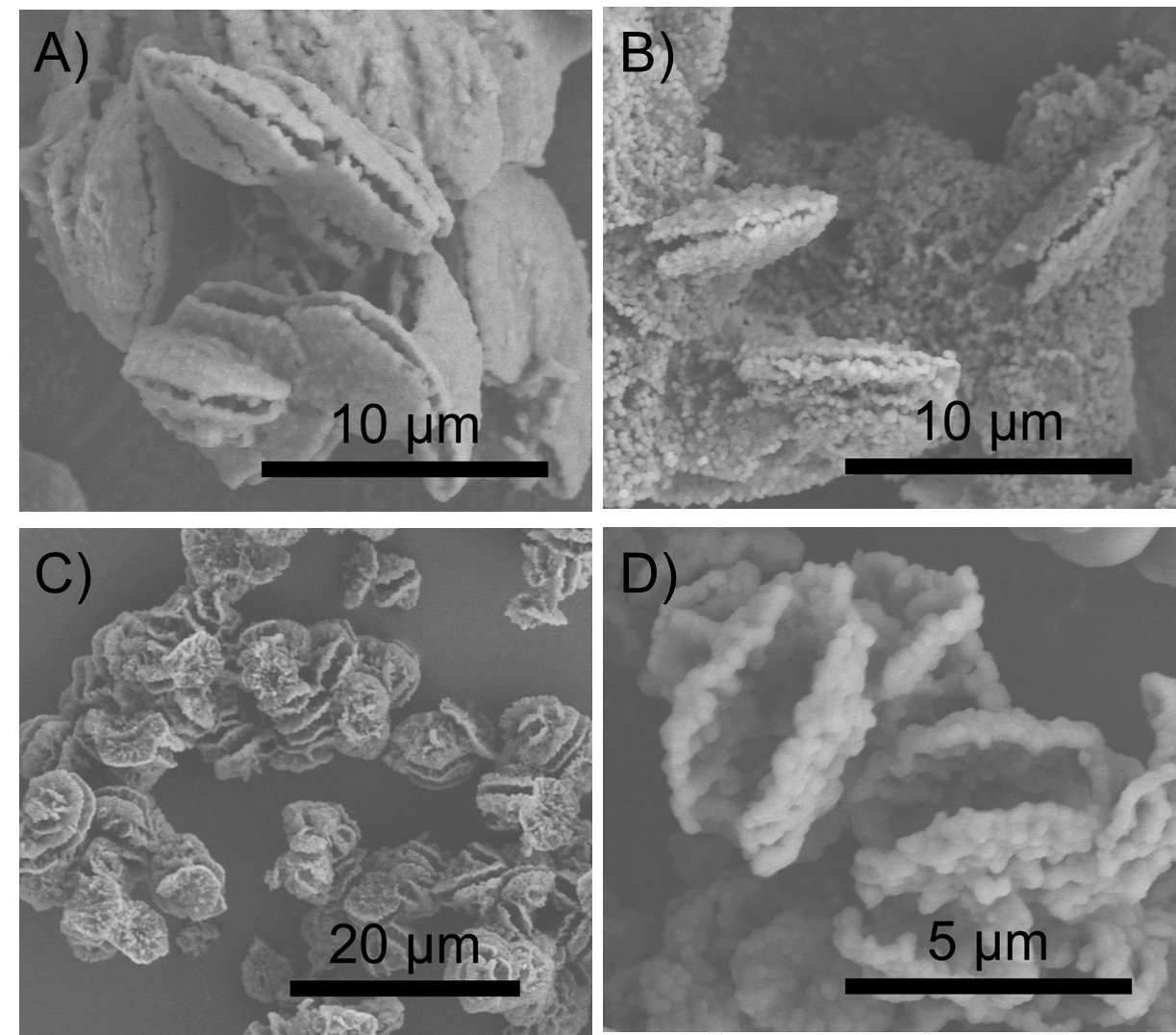


**Fig. S10. SEM images of materials.** (**A**) Arabinose and (**B**) hydrochloric acid replace of xylose and sulfuric acid. (**C, D**) Materials obtained from scale-up experiment.


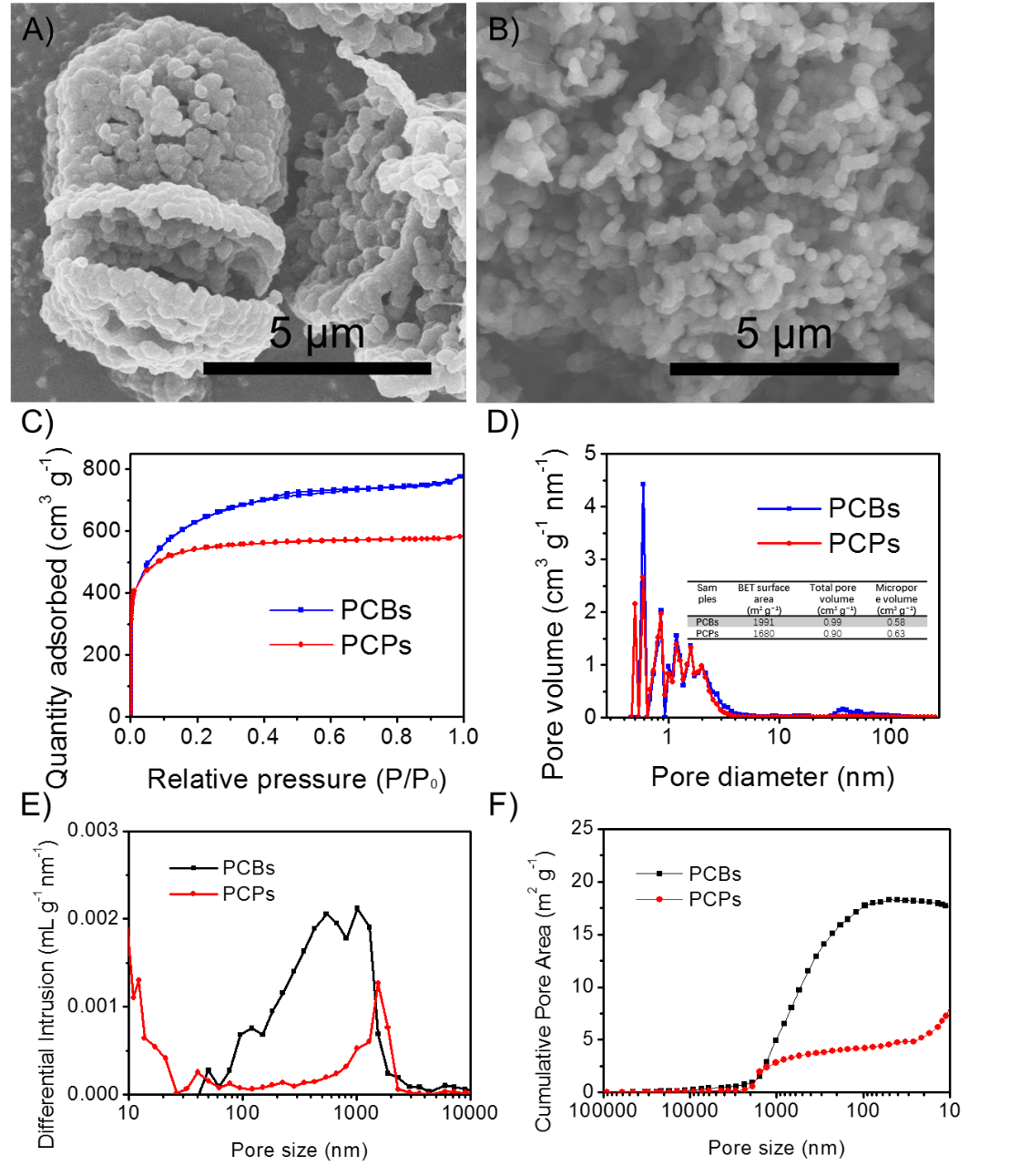


**Fig. S11. Characteristics OF PCBs and PCPs.** (**A**) SEM image of PCBs. (**B**) SEM image of PCPs; N2 adsorption-desorption isotherms and pore size distribution of PCBs and PCPs. (**C**) N2 adsorption-desorption isotherms. (**D**) Pore size distribution; (**E**) Pore volume distributions and (**f**) pore areas for PCBs and PCPs measured by mercury porosimetry.


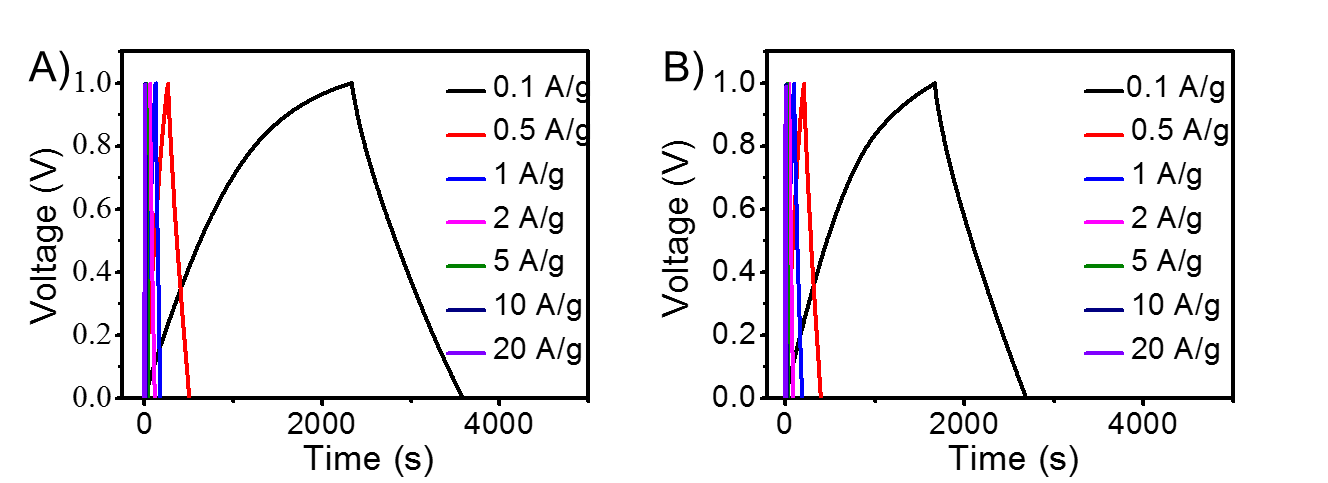


**Fig. S12. GCD curves at various current densities from 0.1 A g^-1^ to 20 A g^-1^.** (**A**) PCBs. (**B**) PCPs.

**Table S1. Summary of the performances of representative porous carbon electrodes from HTC of biomass tested in aqueous electrolyte with a symmetric two-electrode test system.**

| Samlpe | Specific capacitance (F/g) | Capacitance retention ratio (%) | | | | |
| --- | --- | --- | --- | --- | --- | --- |
|  |  | 0.2-2 A/g | 0.5-10 A/g | 0.1-10 A/g | 0.2-20 A/g | 0.5-20 A/g |
| PCBs, this work | 286 (at 0.1 A/g) | / | / | / | 81 | / |
| PCPs, this work | 220 (at 0.1 A/g) | / | / | / | 67 | / |
| hollow carbon nanoflasks, Ref[^26^](#_ENREF_1) | 263 (at 0.1 A/g) | / | 86 | 76 | / | / |
| hierarchical porous flower-like carbons, Ref[^27^](#_ENREF_1) | 200 (at 1.0 A/g) | 84 | / | / | / | / |
| ordered mesoporous carbon, Ref[^28^](#_ENREF_1) | 157 (at 0.5 A/g) | / | / | / | / | 81 |
